# Use of Oral Anticoagulation Therapy in Atrial Fibrillation after Stroke: Results from a Nationwide Registry

**DOI:** 10.1155/2013/601450

**Published:** 2013-11-17

**Authors:** Stine Funder Jespersen, Louisa M. Christensen, Anders Christensen, Hanne Christensen

**Affiliations:** ^1^Department of Neurology, Copenhagen University Hospital Bispebjerg, Bispebjerg Bakke 23, 2400 Copenhagen NV, Denmark; ^2^Department of Radiology, Copenhagen University Hospital Bispebjerg, Bispebjerg Bakke 23, 2400 Copenhagen NV, Denmark

## Abstract

*Background*. The knowledge is still sparse about patient related factors, influencing oral anticoagulation therapy (OAC) rates, in stroke patients with atrial fibrillation (AF). *Aims*. To assess the use of OAC in ischemic stroke patients diagnosed with AF and to identify patient related factors influencing the initiation of OAC. *Methods*. In the nationwide Danish Stroke Registry we identified 55,551 patients admitted with acute ischemic stroke from 2003 to 2011. Frequency analysis was used to assess the use of OAC in patients with AF, and logistic regression was used to determine independent predictors of OAC. *Results*. 17.1% (*n* = 9,482) of ischemic stroke patients had AF. OAC prescription rates were increasing, and in 2011 46.6% were prescribed OAC, 42.5% had a contraindication, and 3.7% were not prescribed OAC without a stated contraindication. Younger age, less severe stroke, and male gender were positive predictors of OAC, while excessive alcohol consumption, smoking, and institutionalization were negative predictors of OAC (*P* values < 0.05). *Conclusions*. Advanced age, severe stroke, female gender, institutionalization, smoking, and excessive alcohol consumption were associated with lower OAC rates. Contraindications were generally present in patients not in therapy, and the assumed underuse of OAC may be overestimated.

## 1. Introduction

Atrial fibrillation (AF) is an important risk factor for ischemic stroke [1, 2]. The detection of either permanent or paroxysmal AF has an immediate impact on treatment and risk reduction in stroke patients [3]. It is well known that oral anticoagulant therapy (OAC), until 2011 exclusively in Denmark with vitamin K antagonists (VKA), is indicated as secondary prevention [4] and lowers the relative risk of recurrent stroke by two thirds [5, 6]. Nevertheless, it has repeatedly been alleged that AF patients do not receive OAC as often as it is clinically indicated [7–10]. It has been suggested that physicians may overestimate bleeding risk from OAC and underestimate its benefits in stroke prevention [11, 12]. However, the knowledge about motivations, including patient related factors, driving clinical decision-making, and thus influencing therapy rates, is still sparse. 

 The aim of study was to assess the use of OAC in ischemic stroke patients diagnosed with AF and to identify patient related factors influencing the initiation of OAC in possible eligible patients, based on the nationwide Danish Stroke Registry, in the period from 2003 to 2011.

## 2. Methods 

### 2.1. The Danish Stroke Registry

The Danish National Health Service provides tax-supported health care for all citizens, including access to hospital care with no costs. All medical emergencies, including stroke, are exclusively treated at public hospitals, and the visitation is based upon catchment's area and diagnoses. The Danish Stroke Registry (DAP), former a part of the Danish National Indicator Project, is a nationwide stroke registry initiated in 2003. DAP monitors and improves the quality of care provided by hospitals by benchmarking. Evidence based standards, indicators, and prognostic factors are used. Data is collected prospectively in all 35 stroke units in the country by health care specialists, in the stroke units, through a mandatory, standardized registration form [13]; audits are performed. These stroke units serve the entire Danish population of approximately 5.5 million inhabitants. The coverage of stroke units is complete, and data on patients with concurrent illnesses causing admission to other departments is also collected systematically. All patients (≥18 years) admitted to Danish hospitals with acute stroke according to the World Health Organization criteria are eligible for inclusion in DAP. Only patients with a final diagnosis of intracerebral haemorrhage, cerebral infarction, and stroke not specified as haemorrhage or infarction are recorded in the registry. Computer tomography (CT) and/or magnetic resonance imaging (MRI) scans on the day of admission are quality indicator as they differentiate cerebral infarction and intracerebral haemorrhage and exclude, for example, subdural hematoma or tumour. In 2011, 85% of all acute stroke patients in Denmark had a CT or MRI scan on the day of admission, and during hospitalization virtually all patients were scanned [14]. Regular, structured audits are conducted nationally, regionally, and locally to ensure data validity of DAP [13]. In 2011 data-completeness (the number of stroke patients in DAP compared to the number of patients with hospital admittance registered in the National Patient Register) was ≥90%, and the number of missing values in DAP was <10% [14]. 

### 2.2. Study Design

All data was provided by DAP. Data regarding the following items were recorded during hospital stay by treating doctors: discharge diagnosis, date of admission, atrial fibrillation (yes/no, regardless if previous, current, paroxysmal, or chronic), and oral anticoagulant therapy (prescribed, not prescribed without stated reason or contraindication present). Until approval of dabigatran etexilate for the Danish market in August 2011, OAC consisted of VKA only, and >95% of VKA was warfarin. Only use of OAC and not the specific product is recorded in the registry; however, the vast majority of patients were prescribed warfarin. OAC in patients with AF within 10 days of stroke onset is a quality indicator. If a patient has AF the treating physician records if OAC is given; is not initiated in spite of existing indication or is considered contraindicated. If considered contraindicated, the cause is indicated by selecting one of the following: Recent surgery, recent bleeding, thrombocyte inhibitor treatment mandatory, uncontrolled hypertension, hemorrhagic diastase, dementia, alcoholism, patient rejecting OAC therapy, pregnancy, moribund patient, high fall risk, previous bleeding, high age, non-compliance, significant liver failure, kidney failure, endocarditis in native valves, and large cerebral infarction with edema and/or high bleeding risk. Data regarding the following patient characteristics included: age, sex, alcohol consumption (≤14/21, >14/21 drinks (5 g alcohol) per week for women and men, resp.), smoking habits (current smoker, occasionally smoker, former smoker, or never-smoker), marital status (cohabiting, living alone, or other), type of residence (private home, nursing home, another form of residence), stroke severity at admission assessed by the Scandinavian Stroke Scale (SSS) score (0–58 points) [15], and a history of the following conditions (yes/no): diabetes mellitus, acute myocardial infarction, hypertension, and previous stroke. CHADS_2_ (Congestive heart failure, Hypertension, Age ≥ 75, Diabetes, Stroke (doubled)) [16], CHA_2_DS_2_-VAS_c_ (congestive heart failure, hypertension, age ≥ 75 (doubled), diabetes, stroke (doubled); vascular disease, age ≥ 65, sex category (female)) [17], or HAS-BLED (hypertension, abnormal renal/liver function, stroke, bleeding history or predisposition, Labile INR (international normalized ratio), elderly (e.g., age > 65, frailty, etc.), and drugs/alcohol concomitantly) [18] scores were not registered.

### 2.3. Study Population

Data was obtained from DAP covering the period from 2003 to 2011. 105,968 patients were included in the registry. Entries dated before 2003 and after 2011 (*n* = 116), entries without admission year (*n* = 19), and patients aged <18 years (*n* = 20) were excluded from analysis. Further analysis was based on patients with a final diagnosis of ischemic stroke, and consequently 55,551 patients remained in the analysis. 9,482 patients with AF were identified and remained in the final analysis.

### 2.4. Statistical Analysis

All statistical analyses were done using IBM SPSS Statistics version 20.0.0. 

 Frequency analysis was used to investigate the use of OAC in patients with ischemic stroke and AF. Frequency analysis was used to describe distribution of OAC status, gender, and stroke severity according to age. Characteristics of patients in therapy and not in therapy were compared using *χ* ^2^-test and Student's *t*-test as appropriate. For categorical variables with more than two categories (marital status, type of residence, alcohol consumption, and smoking habits) binary variables were computed (living alone, institutionalization, excessive alcohol consumption, and smoking). 

 Multiple logistic regression analysis was performed to identify independent predictors of OAC prescription. The variables included were age (categorized into four groups), gender, stroke severity measured by SSS score (categorized into four groups), institutionalization, living alone, smoking, excessive alcohol consumption, and a history of diabetes, acute myocardial infarction, hypertension, or previous stroke. *P* values < 0.05 were considered significant.

 Values not stated were fused with system missing values, included in analysis, and reported as missing. In patient characteristics, missing values were excluded, and the total valid count for each risk factor is reported. 

 The study was approved by the Danish Data Protection Agency, file number 2007-58-0015. 

## 3. Results

### 3.1. Use of Oral Anticoagulation Therapy

Amongst the 55,551 ischemic stroke patients, 9,482 (17.1%) had AF. Of these, 3,938 (41.5%) were prescribed OAC; 3,385 (35.7%) were registered as having a contraindication, whilst 1,459 (15.4%) were not prescribed OAC without a stated reason, and missing values constituted overall 700 (7.4%). The proportion of patients in OAC amongst patients without a contraindication was on average 73.0%, reaching 91.9% in 2011. During the study period the rates of OAC increased from 36.0 to 46.6%; the rates of contraindications increased from 18.9 to 42.5%, whereas the rates of patients not prescribed OAC, without a stated contraindication, decreased during period from 38.5% in 2003 to 3.7% in 2011. The amount of missing values remained the same. It was 6.6% in 2003 versus 7.4% in 2011. 

 Characteristics of the patients in OAC and not in OAC are presented in Table 1. Patients in OAC were younger (mean age 76.5 ± 9.5 years versus 81.7 ± 9.5 years), had less severe strokes indicated by higher SSS score (mean score 43 ± 15 points versus 32 ± 18 points), had less often a history of previous stroke, tended not to live alone, and were more often males (*P* values < 0.05). Patients in OAC were less often institutionalized (*P* value < 0.05). Alcohol consumption and smoking habits, as well as a history of diabetes, hypertension, or acute myocardial infarction did not differ significantly in patients with or without prescription of OAC. 

**Figure 1:**
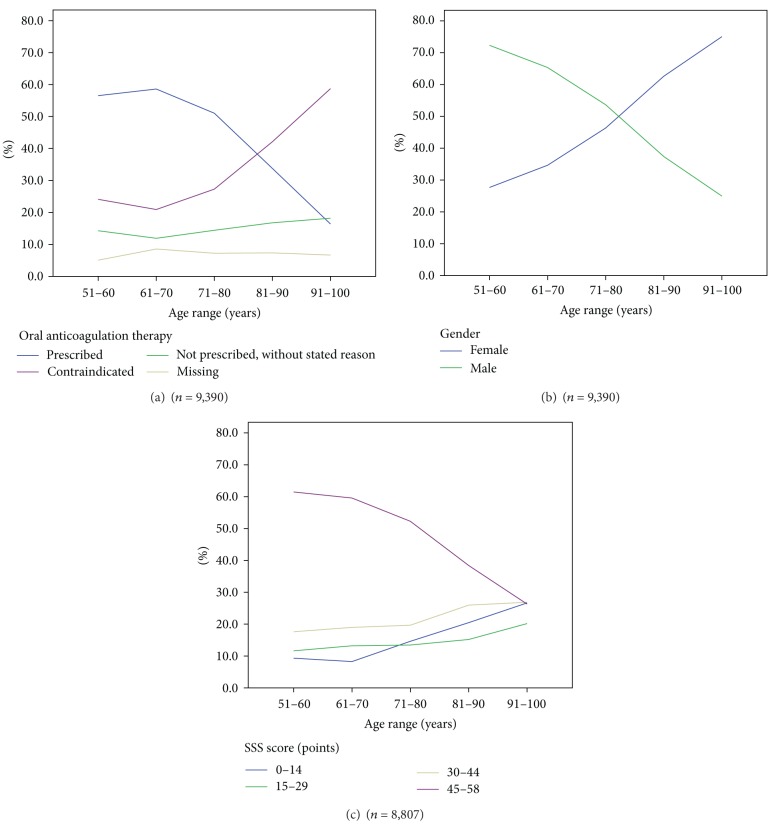
Age distribution of OAC status, gender, and stroke severity in AF patients in the age range of 51–100 years (*n* = 9,390).

 The proportion of patients receiving OAC was decreasing with age, from 56.5% in the age range 51–60 years to 16.4% in the age range 91–100 years. The proportion of patients with contraindications showed the opposite trend and increased with age, from 24.1% in the age range 51–60 years to 58.7% in the age range 91–100 years. The proportion of not prescribed without stated reason and missing values were only slightly increasing with age from 14.3% and 5.1% in the age range 51–60 years to 18.2% and 7.3% in the age range 91–100 years, respectively (Figure 1(a)). Amongst ischemic stroke patients with AF there were more females (5,080) than males (4,402), and the proportions varied markedly according to age. The proportion of females increased with age, from 27.7% in the age range 51–60 years to 75% in the age range 91–100 years (Figure 1(b)). The stroke severity markedly increased with age, measured by decreasing SSS score. The proportion of mild strokes decreased with age, with a corresponding increase in moderate, severe, and very severe strokes (Figure 1(c)).

### 3.2. Independent Predictors of Oral Anticoagulation Therapy

In the logistic regression model relatively younger age, less severe stroke, and male gender were significant positive predictors of OAC prescription, whereas institutionalization, smoking, and excessive alcohol consumption were significant negative predictors of OAC prescription (*P* values < 0.05) (Table 2). Compared to the oldest age group (>84 years), likelihood of OAC prescription was almost doubled (OR = 1.984) in the age group 75–84 years, and further increased (OR = 2.780) in the age group 65–74 years which had similar odds (OR = 2.721) as the youngest age group (<65 years). The likelihood was steadily increasing with decreasing stroke severity (OR = 1.917; 2.468; 3.881, resp.). Male gender was a weak positive predictor (OR = 1.197). Being institutionalized was associated with more than halving the likelihood of receiving OAC (OR = 0.430). Smoking was a weak negative predictor (OR = 0.871) and excessive alcohol consumption was a moderate negative predictor (OR = 0.692). Living alone, or having a history of diabetes, acute myocardial infarction, hypertension or previous stroke were not independent predictors of OAC. Patients with missing values were excluded from analysis; 4,165 (43.9%) had at least one missing value, and 5,317 (56.1%) patients were included in the final binary logistic regression.

## 4. Discussion

### 4.1. Principal Findings

In the present study positive predictors of OAC were relatively younger age, less severe stroke, and male gender, while negative predictors were excessive alcohol consumption, smoking, and institutionalization. Age was one key factor influencing OAC decision. On average 41.5% of all patients admitted from 2003 to 2011 with ischemic stroke and AF were prescribed OAC. A majority of patients not in OAC had a contraindication.

### 4.2. Relations to Other Studies

OAC was administered in 41.5% of ischemic stroke patients with AF, in conformity with results from a nationwide Swedish registry (Riks-Stroke), where 11% received primary prevention and 33.5% received secondary prevention [19] and in line with an American study of medical records, where 42% of AF patients were receiving warfarin [8]. In Stockholm Cohort-Study 54% of AF patients without contraindications received warfarin [7], which is a smaller proportion than the equivalent (73%) in the present study. However, the study population in Stockholm Cohort-Study was different from ours, since not all patients had ischemic stroke, but other risk factors, as an indication for OAC. We speculate that the increased focus on atrial fibrillation during period, as well as the quality indicator system which directly motivates the treating clinicians to follow guidelines, may partly explain the high rate (73%) of patients in relevant OAC. 

 As it is well known from previous studies, we found that stroke severity increased with age [20, 21]. Female gender was overrepresented in ischemic stroke patients with AF. Male gender was more frequent in younger age groups, whereas female gender was dominating in the oldest patients, in accordance with that female gender and advanced age are reported to increase stroke risk in AF patients [4]. 

In our model the strongest predictor of OAC prescription was stroke severity, with higher SSS score associated with higher odds; the more severe the stroke was the less likely the patient was to be receiving OAC. This is in conformity with the finding in Riks-Stroke that being fully conscious on admission was associated with OAC prescription [19]. In accordance with Riks-Stroke, male gender was a weak predictor and relatively younger age was a strong predictor of OAC [19]. Being institutionalized was more than halving the likelihood of receiving OAC. This is comparable to Riks-Stroke's finding that patients with independent ADL functions before the stroke, and patients being discharged to home rather than institution, were more likely to receive OAC [19]. Nursing home residents are presently often severely disabled and with significant cognitive deficits, as home care offers extensive help in own home. Institutionalized patients will consequently often be high risk patients if treated with OAC and the potential benefit often will be estimated as small by treating physicians and relatives.

 In our model comorbidity such as hypertension and diabetes did not predict OAC, in line with Stockholm Cohort-Study [7]. Excessive alcohol consumption was a negative predictor of OAC, as anticipated, since it is considered a relative contraindication. Smoking was a negative predictor, however, statistically weak.

### 4.3. Strengths and Weaknesses

The strengths of the present study include a population-based design that comprises the entire Danish population and reflects routine clinical practice. Health care specialists collected detailed quality data prospectively, which minimizes the risk of selection and information bias. Minimal exclusions were made, since the exclusions of patients with missing data potentially would have introduced selection bias. Risk of referral bias was low, since it can be assumed that all patients with acute symptoms of stroke are referred to the public health care system if hospitalized. Traditionally hospitalization rates are high after stroke in Denmark [22].

 The weaknesses of the present study include the retrospective design and the risk of misclassifications during data collection in routine clinical settings. Some change of practice may have occurred during the 9-year period. However, participation in DAP is mandatory for all departments treating patients with acute stroke, and extensive efforts are made to ensure data validity. Missing values in some items were decreasing during study period, reflecting better implementation of DAP. Although missing data should always be a reason for concern, we have no reason to believe these substantially influenced results, since the missing data were incidental; however, in some analyses this caused substantial exclusion of patients. Further, more detailed data on patients for example, CHA_2_DS_2_-VAS_c_ score, might have contributed to a more detailed interpretation of our findings; however, these data were not collected.

### 4.4. Perspective

The present study stresses the importance of continued attention to therapy rates. However, it also raises the question if alleged underuse of OAC really is of the previously assumed size or could for a majority be explained by contraindications and risk/benefit ratios made by clinicians on an individual patient level. When the decision about OAC is made it is important to be knowledgeable about present guidelines and actual contraindications, especially when treating older patients, patients with severe strokes, or institutionalized patients, which may frequently lead to not treating the patient. Novel OAC (NOAC) drugs such as dabigatran, rivaroxoban, and apixaban show efficacy and safety at least comparable to warfarin [23–26]. It is difficult to predict if NOAC will further increase the rate of anticoagulation after stroke, though efficacy and safety profiles are non-inferior and most likely slightly better than warfarin [24–26]; these drugs also have limitations and the trials did not include old patients with reduced independency in daily living. Especially dabigatran etexilate is almost exclusively excreted by renal pathway, which increases the risk of hemorrhage in case of acute reduction of renal elimination in case of, for example, infection and dehydration. Further, there is no documented antidote to any of the NOACs, whereas pro-thrombin-complex and vitamin K efficiently and fast can revert VKA; however, the effect on clinical outcome in VKA-related intracerebral hemorrhage remains to be documented [27]. NOACs and other options such as self-monitored warfarin [28] as alternatives to traditional anticoagulation practice will improve the options of individualizing treatment and might thereby contribute positively to patient adherence. 

## 5. Conclusion

In summary, relatively younger age and less severe stroke were strong positive predictors of OAC. Smoking and excessive alcohol consumption as well as being institutionalized were negative predictors of OAC. Male gender was a weak but significant positive predictor. Contraindications were generally present in patients not in therapy. Underuse of OAC may be overestimated and represents risk/benefit estimates in individual high-risk patients. 

## Figures and Tables

**Table 1:** Characteristics of ischemic stroke patients with AF (*n* = 8,782) in and not in OAC.

Characteristics	OAC (*n* = 3,938)		No OAC (*n* = 4,844)		Total	*P* value
	Mean (SD)	*n* (%)	Mean (SD)	*n* (%)		
Age (years)	76.5 (±9.5)		81.7 (±9.5)		8,782	<0.0001
SSS score (points)	43 (±15)		32 (±18)		8,272	<0.0001
Gender						
Female		1,812 (46.0)		2,888 (59.6)	8,782	<0.0001
Male		2,126 (54.0)		1,956 (40.4)		
Institutionalized						
Yes		193 (5.1)		792 (17.3)	8,354	<0.0001
No		3,591 (94.9)		3,778 (82.7)		
Previous stroke						
Yes		1,174 (30.7)		1,572 (34.0)	8,447	0.001
No		2,656 (69.3)		3,045 (66.0)		
Living alone						
Yes		1,629 (42.5)		2,562 (55.2)	8,476	<0.0001
No		2,205 (57.5)		2,080 (44.8)		
Smoking						
Yes		799 (24.8)		805 (23.6)	6,635	0.232
No		2,420 (75.2)		2,611 (76.4)		
Excessive alcohol consumption						
Yes		161 (4.7)		184 (5.0)	7,077	0.581
No		3,244 (95.3)		3,488 (95.0)		
Diabetes mellitus						
Yes		620 (16.1)		759 (16.2)	8,521	0.856
No		3,230 (83.9)		3,912 (83.8)		
Acute myocardial infarction						
Yes		553 (14.7)		687 (15.4)	8,226	0.431
No		3,200 (85.3)		3,786 (84.6)		
Hypertension						
Yes		2,247 (59.8)		2,679 (59.3)	8,278	0.668
No		1,513 (40.2)		1,839 (40.7)		

**Table 2:** Multiple logistic regression analysis of treatment with OAC in ischemic stroke patients with AF (*n* = 9,482).

	*P* value	OR	95% CI
Age group (years)			
>84	Reference category		
75–84	<0.0001	1.984	1.716–2.293
65–74	<0.0001	2.780	2.326–3.321
<65	<0.0001	2.721	2.170–3.413
SSS group (points)			
Very severe (0–14)	Reference category		
Severe (15–29)	<0.0001	1.917	1.502–2.447
Moderate (30–44)	<0.0001	2.468	1.982–3.074
Mild (45–58)	<0.0001	3.881	3.170–4.751
Male gender	0.004	1.197	1.058–1.354
Institutionalization	<0.0001	0.430	0.337–0.548
Smoking	0.053	0.871	0.756–1.002
Excessive alcohol consumption	0.007	0.692	0.528–0.906
